# Gut mucosal immune responses and protective efficacy of oral yeast Cyprinid herpesvirus 2 (CyHV-2) vaccine in *Carassius auratus gibelio*

**DOI:** 10.3389/fimmu.2022.932722

**Published:** 2022-07-29

**Authors:** Zhao-Ran Dong, Qing-Jiang Mu, Wei-Guang Kong, Da-Cheng Qin, Yong Zhou, Xin-You Wang, Gao-Feng Cheng, Yang-Zhi Luo, Tao-Shan Ai, Zhen Xu

**Affiliations:** ^1^ Department of Aquatic Animal Medicine, College of Fisheries, Huazhong Agricultural University, Wuhan, China; ^2^ State Key Laboratory of Freshwater Ecology and Biotechnology, Institute of Hydrobiology, Chinese Academy of Sciences, Wuhan, China; ^3^ Yangtze River Fisheries Research Institute, Chinese Academy of Fishery Sciences, Wuhan, China; ^4^ Wuhan Chopper Fishery Bio-Tech Co., Ltd, Wuhan Academy of Agricultural Science, Wuhan, China; ^5^ Laboratory for Marine Biology and Biotechnology, Qingdao National Laboratory for Marine Science and Technology, Qingdao, China

**Keywords:** CyHV-2, *gibel carp*, yeast surface display, oral vaccination, protective efficacy, mucosal immune response

## Abstract

Cyprinid herpesvirus 2 (CyHV-2) causes herpesviral hematopoietic necrosis (HVHN) disease outbreaks in farmed Cyprinid fish, which leads to serious economic losses worldwide. Although oral vaccination is considered the most suitable strategy for preventing infectious diseases in farmed fish, so far there is no commercial oral vaccine available for controlling HVNN in gibel carp (*C. auratus gibelio*). In the present study, we developed for the first time an oral vaccine against CyHV-2 by using yeast cell surface display technology and then investigated the effect of this vaccine in gibel carp. Furthermore, the protective efficacy was evaluated by comparing the immune response of a single vaccination with that of a booster vaccination (booster-vaccinated once 2 weeks after the initial vaccination). Critically, the activities of immune-related enzymes and genes expression in vaccine group, especially in the booster vaccine group, were higher than those in the control group. Moreover, strong innate and adaptive immune responses could be elicited in both mucosal and systemic tissues after receipt of the oral yeast vaccine. To further understand the protective efficacy of this vaccine in gibel carp, we successfully developed the challenge model with CyHV-2. Our results showed the relative percent survival was 66.7% in the booster vaccine group, indicating this oral yeast vaccine is a promising vaccine for controlling CyHV-2 disease in gibel carp aquaculture.

## Introduction

Gibel carp (*Carassius auratus gibelio*) is an important freshwater fish species that is cultured worldwide, especially in China, which has great market value and development potential (1). According to recent statistics, the total production of gibel carp in China exceeded 2.7 million tons in 2020, accounting for approximately 9% of the national freshwater fish breeding output and ranking fifth among all cultured species (2). Cyprinid herpesvirus 2 (CyHV-2) is a linear double-stranded DNA virus that belongs to the genus *Cyprinivirus* (family Alloherpesviridae). This virus is a fatal contagious aquatic pathogen that affects cyprinid fish such as ornamental goldfish (*Carassius auratus*), crucian carp (*C. carassius*), and gibel carp (3). The clinical signs of CyHV-2 infection include lethargy and inappetence, gill bleeding, massive abdominal hemorrhage, abdominal swelling, and eyeball protrusion (4). Herpesviral hematopoietic necrosis (HVHN) caused by CyHV-2 infection was first reported in goldfish in Japan in 1992, with mortality rates reaching up to 90%-100% (5). Since then, CyHV-2 has rapidly spread across the globe, and CyHV-2 infection has been responsible for huge economic losses to the cyprinid fish aquaculture industry, especially to gibel carp farms in China (6). Effective drugs against CyHV-2 infection have not yet been identified, and therefore there is a pressing need to develop effective prevention and treatment strategies for HVHN disease.

Vaccination has been shown to be the most effective countermeasure against viral infection due to its immunostimulatory effect, thus reducing morbidity and mortality. Experimental studies on CyHV-2 vaccines began in 2013 (7), and several vaccines have since been developed and used to prevent and control CyHV-2 infection, including inactivated vaccines, live vaccines, DNA vaccines, and subunit vaccines (7–12). Particularly, live vaccines are the closest alternative to natural pathogen infection, in addition to being powerful and eliciting durable immune responses (10). The two main types of live vaccines are attenuated and recombinant-vectored vaccines. Live attenuated vaccines are commercially available and authorized to be used in the aquaculture industry (13). However, live attenuated vaccines carry a risk of reverting to their wild-type form and causing disease (14). Unlike live pathogens or DNA vaccines, subunit vaccines do not pose the risk of invading the host or integrating with the host DNA (15). With the development of gene recombination technology, safer alternatives to live recombinant vector vaccines have begun to emerge in recent years. In fish vaccinology, the most widely used protein expression systems for the production of subunit antigens are *E. coli* and yeast. Despite the high productivity exhibited by *E. coli*, the antigens must need to purify to eliminate endotoxin before safe use, and they often produce insoluble, misfolded, and nonfunctional proteins (16). Using whole *Saccharomyces cerevisiae* (*S. cerevisiae*) cells expressing vaccine antigens can yield low-cost vaccines as purification is not required (17).

So far, three major routes: injection, oral, and immersion are most widely used in fish vaccination. Unfortunately, most currently available vaccines against CyHV-2 are administered through injection, which is not only stressful to the fish but also labor-intensive and costly, thus limiting their use and promotion (13). Apart from the above-mentioned limitations, the mucosal immune response cannot be effectively stimulated by injection vaccination (18). Anal and nasal vaccination have been tested in the laboratory with good results, but their practical application is doubtful because of the associated difficulty of administration (19, 20). In immersion or bath vaccines, the main target tissues are the mucosal surfaces of the skin, gills, and even nasopharynx. The main concerns for bath immunization remain the large amounts of antigen required and the immunization efficiency. Similar to mammals, the teleost fish intestine is among the most important mucosal immune organs because it constitutes a key entry point for a wide variety of pathogens (21). Oral vaccination can directly deliver the vaccines to the gut mucosal surface and can stimulate mucosal and systemic immune responses against infections (15). Moreover, oral vaccines are well suited for fish immunization because they eliminate the need to handle animals and can be easily applied to both small and large sized fish, in addition to being far less labor-intensive than injection (13).

An ideal vector and pathogen gene with immunogenic activity should be considered before the preparation of a recombinant vaccine. Due to its fully sequenced genome, natural adjuvant properties, and food-grade safety, *S. cerevisiae* is often considered an ideal model vector of vaccines for clinical or veterinary use. The utilization of yeasts for the synthesis of protein antigens from pathogenic species is a novel strategy for the development of recombinant vector vaccines, and this approach is particularly well-suited for the development of oral vaccines (22). Functional prediction of the whole genome of CyHV-2 (GenBank No.NC_019495.1) identified 36 open reading frames (ORFs) encoding for the membrane proteins. Among them, ORF25, ORF25B, and ORF146, contained the immunoglobulin-like domains and possibly had immunogenicity (23). Yuan et al. analyzed the immunogenicity based on the positive serum reaction between the encoding CyHV-2 membrane protein expression products and CyHV-2. The screening revealed that of the four candidate proteins (i.e., ORF16, tORF25, tORF64, and ORF146), which gave a positive serum reaction with CyHV-2 used for the immunization of gibel carp, the antibody titer induced by tORF25 was the highest (12). Additionally, Zhou et al. have demonstrated CyHV-2 ORF25 is an ideal candidate for the development of vaccines against CyHV-2 due to its high immunogenic activity (9). Compared with the intracellular expression of recombinant viral proteins, display of viral proteins on the surface of vector cells may facilitate their recognition by the host mucosal immune system, thereby enhancing their ability to induce protective immunity (24). Yeast surface display is a whole-cell platform used for the heterologous expression of proteins immobilized on the yeast’s cell surface (25). Previous studies have shown that feeding yeast particles expressing the antigen protein from the virus on their surface protects pigs against viral infections (26, 27). Therefore, additional studies are needed to develop novel vaccines against CyHV-2 infection based on the yeast surface display system.

Here, we prepared an oral vaccine using the yeast surface display system to express the ORF25 gene from CyHV-2 on *S. cerevisiae*. After vaccination and booster vaccination of gibel carp, we evaluated the safety and immunogenicity of the oral yeast vaccine by evaluating cumulative mortality, gut histology, body fluid biochemical indices, immune-related gene expression, and pathogen loads. Finally, a challenge test was performed to confirm the protective efficacy of the yeast vaccine against CyHV-2. Taken together, our results indicate that the orally-administered live recombinant yeast vaccine developed herein is a promising candidate for the control of CyHV-2 in the gibel carp farming industry. Additionally, our findings provide crucial insights into the role of oral vaccination in combating viral infections in teleost fish.

## Materials and methods

### Construction of an *S. cerevisiae-*based vaccine

To generate pYD1-ORF25, the artificial sequence of the ORF25 gene from the CyHV-2 (NC_019495.1) was synthesized. The obtained fragment was inserted into pYD1 (Invitrogen, USA) by the *Eco*RI and *Not*I double digestion, generating the pYD1-ORF25. Positive clones of pYD1-ORF25 were confirmed by PCR detection with primer pairs P1/P2 (P1:5’-GCCAACGATACCGTCAAGGA-3’, P2:5’-TGCGAATATGTCGGCTTGGT-3’). The resulting plasmid pYD1-ORF25 was electroporated into competent *S. cerevisiae* EBY100 cells according to the manufacturer’s instructions and cultured in yeast extract peptone dextrose adenine (YPDA) medium. The positive transformants were cultured in YNB-CAA medium (0.67% yeast nitrogen base without amino acids and ammonium sulfate, 0.5% casamino acids hydrolysate) containing 2.0% galactose. After 60 h induction, the yeast cells were collected by centrifugation at 4000 g for 8 min and stored at 4°C for subsequent assays.

### Immunofluorescence analysis

EBY100/pYD1-ORF25 pellets were incubated with 1:1000 diluted mouse anti-His antibody (ABclonal, China) for 1 h. After washing three times with PBS, Cy3-conjugated AffiniPure goat anti-mouse IgG (Invitrogen, USA) at a dilution of 1:500 was added and incubate at RT for 40 min, then washed three times and resuspended in 500 μl of sterile PBS. Stained bacteria were cytospinned on glass slides and mounted with fluorescent microscopy mounting solution. Images were captured using an Olympus BX53 fluorescence microscope and analyzed with the iVision-Mac scientific imaging processing software (Olympus, Japan).

### Fish maintenance

Healthy gibel carp (25 ± 5 g) used in this study were obtained from a fish farm in Hu Zhou (Zhejiang province, China), and were maintained in aquarium tanks using a water recirculation system. All fish were acclimatized for at least 2 weeks at 25 ± 1°C and fed commercial pellets twice a day before vaccination. Animal procedures were approved by the Animal Experiment Committee of Institute of Hydrobiology, Chinese Academy of Sciences and carried out according to the relative guidelines.

### Fish vaccination

300 gibel carp were randomly divided into three groups (control, vaccine, and booster vaccine), with 100 fish for each. The induced EBY100/pYD1-ORF25 was centrifuged at 2000 g for 10min, and the supernatant was discarded. The yeast vaccine was prepared by mixing the precipitate (6 × 10^-2^ CFU g^-1^) with gibel carp feed with a mass ratio of 1:100. Use gelatinized starch as a binder and add a little water to mix the vaccine with feed thoroughly. The control group was only fed with F3 commercial fish floating feed pellets (Charoen Pokphand, China). The vaccine group was fed with 1.3mg/fish of vaccines and completed within three days. The booster vaccine group received a second vaccination at 18 days in the same way as the first vaccination. The water temperature was controlled at 25 ± 1°C during vaccination.

### Sampling

**Figure 1:**
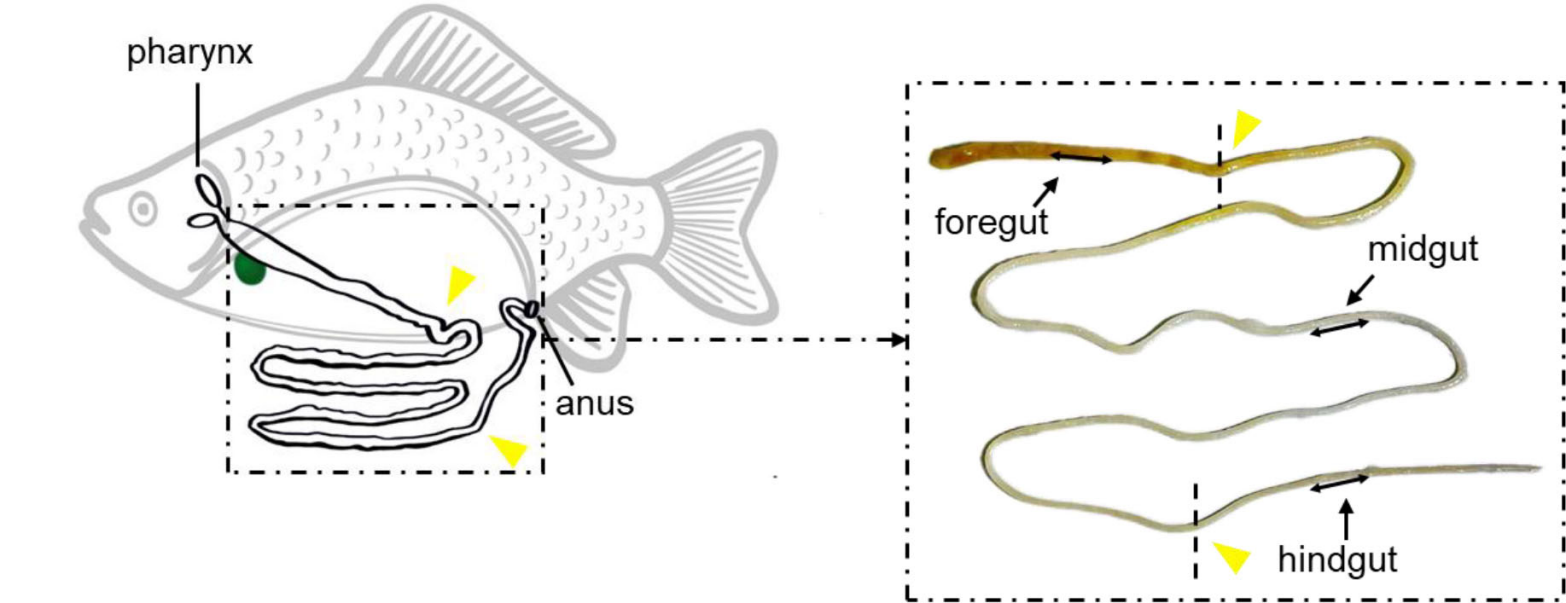
The sampling position of the gibel carp’s gut. Black line with double arrowheads demonstrates the exact location of foregut, midgut, and hindgut sampling in this study. Yellow triangles indicate the dividing line of the foregut, midgut, and hindgut.

Before sampling, gibel carp were anesthetized with MS-222 (Tricaine methanesulfonate, Sigma, USA) at a concentration of 100 mg/l. Blood samples were obtained from the caudal vein and were placed at 4°C overnight, after which were centrifuged at 5000 g for 10 min. The serum was collected and stored at -80°C prior to use. For intestinal mucus collection, the gut of gibel carp was excised and opened longitudinally. The foregut, midgut, and hindgut were distinguished as shown in **Figure 1**, and 0.5 ml 0.9% sodium chloride solution was added onto its surface. The mucosal fluid was gently scraped from the gut’s inner surface, transferred to an Eppendorf tube, and then blew it repeatedly through the injector. The sample was centrifuged at 400 g for 10 min to remove large particles and gibel carp cells. The supernatant was thereafter centrifuged at 10,000 *g* for 10 min. The resulting supernatant was harvested, filtered with a 0.45 μm syringe filter, and stored immediately at -80°C. Tissue and fluids samples from six fish of the control and vaccine groups were taken at 10, 17, 27, and 34 days, and booster vaccine group were taken at 27 and 34 days. Tissue samples were used to evaluate morphological change by H&E and measure the expression of the immune-related genes using quantitative real-time PCR (qPCR), respectively. Intestinal mucus was used to detect the biochemical indexes. The mortalities and clinical signs were daily observed in the vaccine and booster vaccine groups for 40 days.

### Histology and light microscopy studies

The gut tissues for histological examination were dissected and fixed immediately in 4% neutral buffered formalin at room temperature for 24 h, dehydrated, paraffin-embedded, and sectioned. Thereafter, 5 µm sections were stained with hematoxylin and eosin (H&E) prior to examination by light microscopy. Images were acquired in a BX53 microscope (Olympus, Japan) using the Axiovision software. The length-width ratios of the foregut, midgut, and hindgut intestinal folds were measured for evaluating microscopic pathological changes. Parameters of each image were measured by three different researchers and a mean was taken to reduce random error.

### Body fluids biochemical indexes detection and immune-related gene analysis

Complement 3 (C3), total superoxide dismutase (TSOD), and lysozyme (LZM) of serum and gut mucus were assayed by the corresponding commercial kit protocols (Nanjing Jiancheng Bioengineering Institute, China). All the tissue samples were homogenized in 1ml TRIzol (Invitrogen, USA) using steel beads shaking (60 HZ for 1 min) by the high throughput tissue grinder (WONBIO, China). Total RNA was extracted using TRIzol reagent in accordance with the manufacturer’s instructions. The concentration of extracted RNA was determined by spectrophotometry (Nanodrop ND1000, LabTech), and the integrity of the RNA was determined by 1% agarose gel electrophoresis (Agilent Bioanalyser, 2100). To normalize gene expression levels for each sample, equivalent amounts of total RNA (1,000 ng) were used for cDNA synthesis with the SuperScript first-strand synthesis system (YEASEAN, China) in a 20-µl reaction volume. The synthesized cDNA was diluted three times and then was used as a template for qPCR analysis. The qPCR was performed on a qTOWER3G PCR system (Analytik Jena AG, Germany) by using the EvaGreen 2 × qPCR Master mix (YEASEAN, China) as following conditions: 95°C for 5 min, followed by 40 cycles at 95°C for 10 s and at 58°C for 30 s. The change in transcription of genes was calculated as relative fold expression by the 2^-ΔΔCt^ method (28). Moreover, based on performance and stability, the *β-actin* was validated and used as a control gene for the normalization of expression of each single target used in the present study. The results were obtained from three independent experiments, and each was performed in triplicate. The primers used for qPCR are listed in **Supplementary Table S1**.

### Challenge test

CyHV-2 were kind gifts from Pro. Yong Zhou, Yangtze River Fisheries Research Institute of Chinese Academy of Fishery Sciences, China (9). The healthy gibel carp was infected with the virus stock by injection for the virus reactivation. After 3 days, the hepatopancreas, spleen, and kidney mixture (~ 0.5 g) dissected from the diseased fish infected with CyHV-2 was homogenized and mixed with 10ml of PBS. After centrifugation at 8000 g for 15 min, the supernatant was collected and used as CyHV-2 stock. At 41 days post vaccination (dpv), fish from control, vaccine, and booster vaccine groups were randomly divided into two groups, respectively, with 30 fish in each group. Then one group was injected with CyHV-2 stock (100 μl per fish) at the base of the pectoral fin, while the other control group was injected with PBS. Thereafter, at 4 days after challenge (45 dpv), six fish (each group) of these six groups (including: Con [control fish + PBS], Va [vaccinated fish +PBS], Bo-va [booster-vaccinated fish + PBS], Con+Cha [control fish + CyHV-2], Va+Cha [vaccinated fish + CyHV-2], Bo-va+Cha [booster-vaccinated fish + CyHV-2]) were randomly selected and analyzed for pathological changes of gut. The clinical signs, cumulative mortality, and survival rates were recorded daily for 30 days post challenge (dpc) and the relative percent survival (RPS) by the following formula: RPS = (1 – the ratio of mortality percent in the vaccinated group to in the control group) × 100%.

### Standard curve for CyHV-2

Viral nucleic acids were extracted from the hepatopancreas, spleen, and head kidney of fish, with six fish in each group, following the DNA Extraction Kit (QIAGEN, Germany) procedure. A 366 bp coding region of CyHV-2 the DNA helicase gene (AY939867) was amplified by PCR and cloned into pMD19T vector for the construction of recombinant plasmid. After identifying and confirming with PCR reaction, 10-fold serial dilutions of recombinant plasmid were used as standard templates for TaqMan real-time PCR to quantify the virus genomic copy number and generate a standard curve (**Supplementary Figure 2**). The primers used for qPCR are listed in **Supplementary Table S1**.

### Statistical analysis

All data were analyzed using Prism version 8.0 (GraphPad Software). Comparison between two distinct groups was determined using an unpaired Student’s *t*-test. One-way ANOVA with Bonferroni correction was used for multiple comparisons: among groups at each time point. Data are expressed as mean ± SEM. A value of *p* < 0.05 was considered statistically significant.

## Results

### Expression analysis of CyHV-2-ORF25 protein of *S. cerevisiae* EBY100

**Figure 2:**
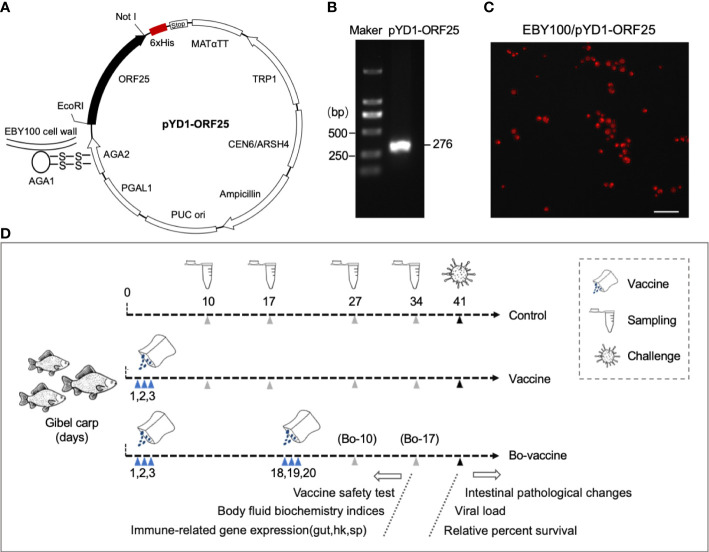
Schematic diagram of the yeast vaccine and experimental strategy. **(A)** pYD1-ORF25 recombinant plasmids displayed on the yeast cell surface through attachment of Aga2 to Aga1 *via* disulfide bonds. **(B)** Positive clones of pYD1-ORF25 identification by agarose gel electrophoresis. **(C)** Immunofluorescence assay of EBY100/pYD1-ORF25 fusion protein Expression. Scale bars, 20 μm. **(D)** Strategy to vaccinate gibel carp. Briefly, vaccine and bo-vaccine fish were orally administered with CyHV-2 yeast vaccine at 1, 2, and 3 days (vaccine) and 1, 2, 3, 18, 19, and 20 days (booster vaccine), respectively. Take samples at the 10, 17, 27, and 34 days to test the safety of the yeast vaccine (cumulative survival after vaccination and morphological changes of gut), body fluid biochemistry indices, and immune-related gene expression. Challenge with CyHV-2 at the 41 days to test intestinal pathological changes, viral load, and relative percent survival.

The yeast display system depended on the a-agglutinin receptor which consisted of two subunits, *aga1* and *aga2*, harboring the capacity of fusion with heterologous protein *via* the GS linker. The Aga2-ORF25 was bound to Aga1 through two disulfide bonds, resulting in the ORF25 display on the yeast wall (**Figure 2A**). The recombinant plasmid pYD1-ORF25 was first confirmed by PCR and the DNA bands of the predicted sizes, indicating that it was successfully constructed (**Figure 2B**). Immunofluorescence assay was used to identify the expression of CyHV-2-ORF25 protein in EBY100/pYD1. We found that red fluorescence was distributed homogeneously on the surfaces of yeast (**Figure 2C**). These results demonstrated that the ORF25 could be expressed on the surface of EBY100.

### Safety evaluation of the yeast vaccine

**Figure 3:**
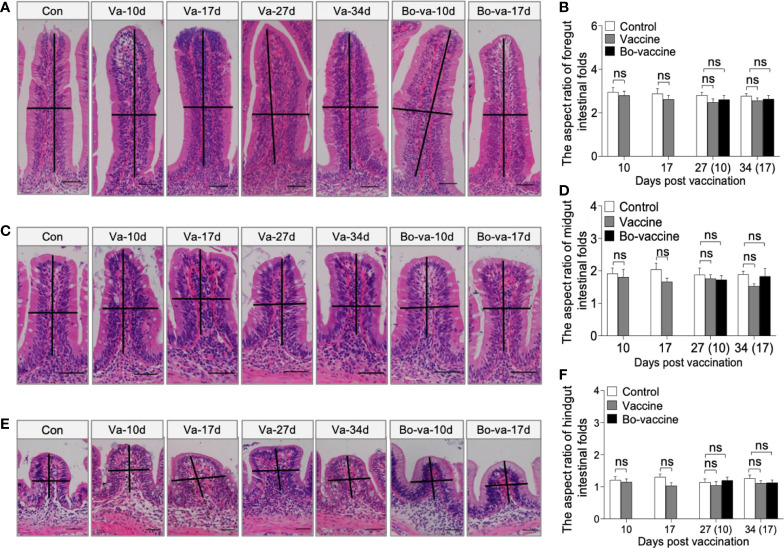
Safety evaluation of oral administration of CyHV-2 yeast vaccine. **(A, C, E)** Histological examination by H&E staining of foregut **(A)**, midgut **(C)**, and hindgut **(E)** from Control fish and Vaccine fish and Bo-vaccine fish (*n* = 6 fish per group). Scale bars, 50 μm. Con, control; Va, vaccine; Bo-va, booster vaccine. **(B, D, F)** The length-width ratio of foregut **(B)**, midgut **(D)**, and hindgut **(F)** intestinal folds in control fish, vaccine fish, and booster vaccine fish (*n* = 6 fish per group). Control *vs*. Vaccine and Bo-vaccine, ns, not significant, unpaired Student’s *t*-test (at 10dpv and 17dpv), one-way ANOVA with Bonferroni correction (at 27dpv and 34dpv). Data are representative of three different independent experiments (mean ± SEM).

To test the safety of the yeast vaccine, 30 fish were randomly sampled from each of the three groups (i.e., control, vaccine, and booster vaccine) and monitored for cumulative mortality daily for 40 days, whereas the other fish were sacrificed at 10, 17, 27, and 34 dpv to collect tissue samples (**Figure 2D**). No dead fish in the vaccine and booster vaccine groups were monitored during the 40 days after vaccination. Histological analysis showed that no significant difference in the aspect ratio of foregut, midgut, and hindgut intestinal folds was observed at any of the selected time points among the control group, vaccine group, and bo-vaccine group (**Figures 3A–F**). Taken together, the oral yeast vaccine did not cause mortality or clinical symptoms of the gut, which indicates that the vaccine is safe for gibel carp.

### Body fluid biochemistry indices

**Figure 4:**
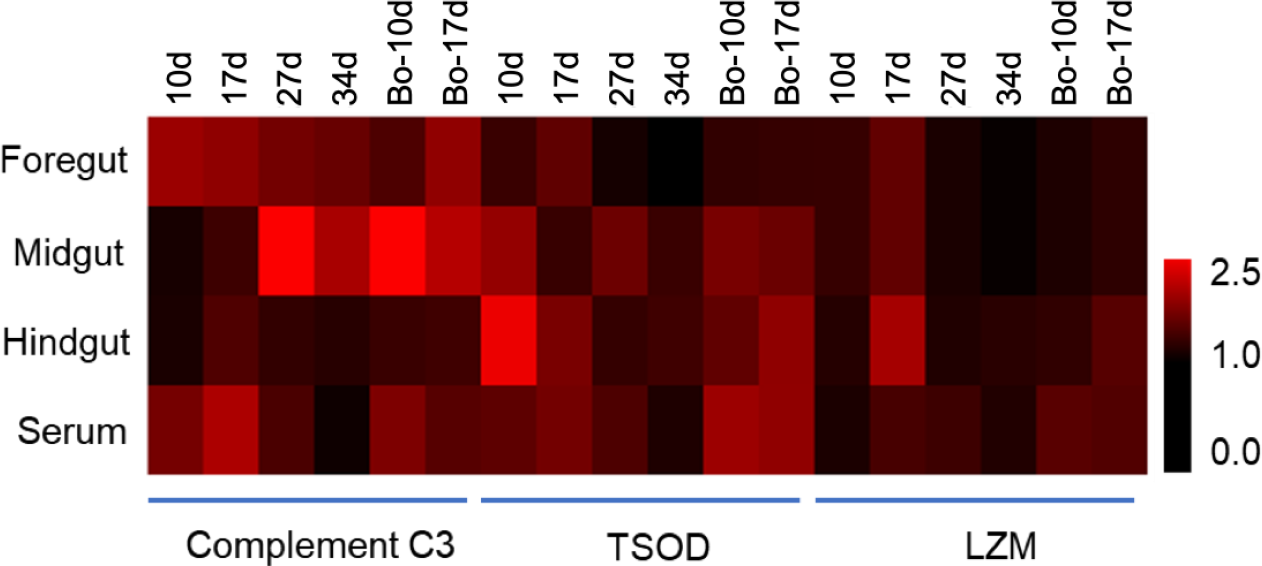
Effects of vaccination on levels of body fluid biochemical parameters of gibel carp. Complement C3, TSOD and LZM enzyme activities in foregut, midgut, and hindgut mucus, as well as serum were detected by using commercial kits (*n* = 6 fish per group). Data are representative of three different independent experiments (mean ± SEM).

The activities of innate immune-related enzymes in foregut, midgut, and hindgut mucus, as well as serum were detected at 10, 17, 27, and 34 dpv using commercial kits (**Figure 4**). Compared to the control group, the C3 activity showed a significant increase in foregut mucus at 10, 17, and 27 dpv, in midgut mucus at 27 and 34 dpv, in hindgut mucus at 17, 27 dpv, and in serum at 10, 17 dpv in the vaccine group. Moreover, we found that the C3 activity in the midgut mucus started to increase later than in the foregut mucus, hindgut mucus, and serum after vaccination. The TSOD activity was significantly higher than that of control fish in the foregut and hindgut mucus at 10 and 17 dpv, in midgut mucus at 10 and 27 dpv, and in serum at 10, 17, and 17 dpv and then returned to normal levels. The LZM activity showed a significant increase in foregut mucus at 10 and 17 dpv, hindgut at 17 dpv mucus, and serum at 17 and 27 dpv, whereas no significant changes were observed in midgut mucus. It is worth noting that the C3, TSOD, and LZM activities either in the gut mucus or serum of the booster-vaccinated fish were slightly higher than that in the vaccine group, suggesting the effect of this oral yeast vaccine administrated twice may be better than once. These results indicated that the oral yeast vaccine could effectively improve the innate immune-related enzyme activities in body fluids.

### Immune-related gene expression after vaccination

**Figure 5:**
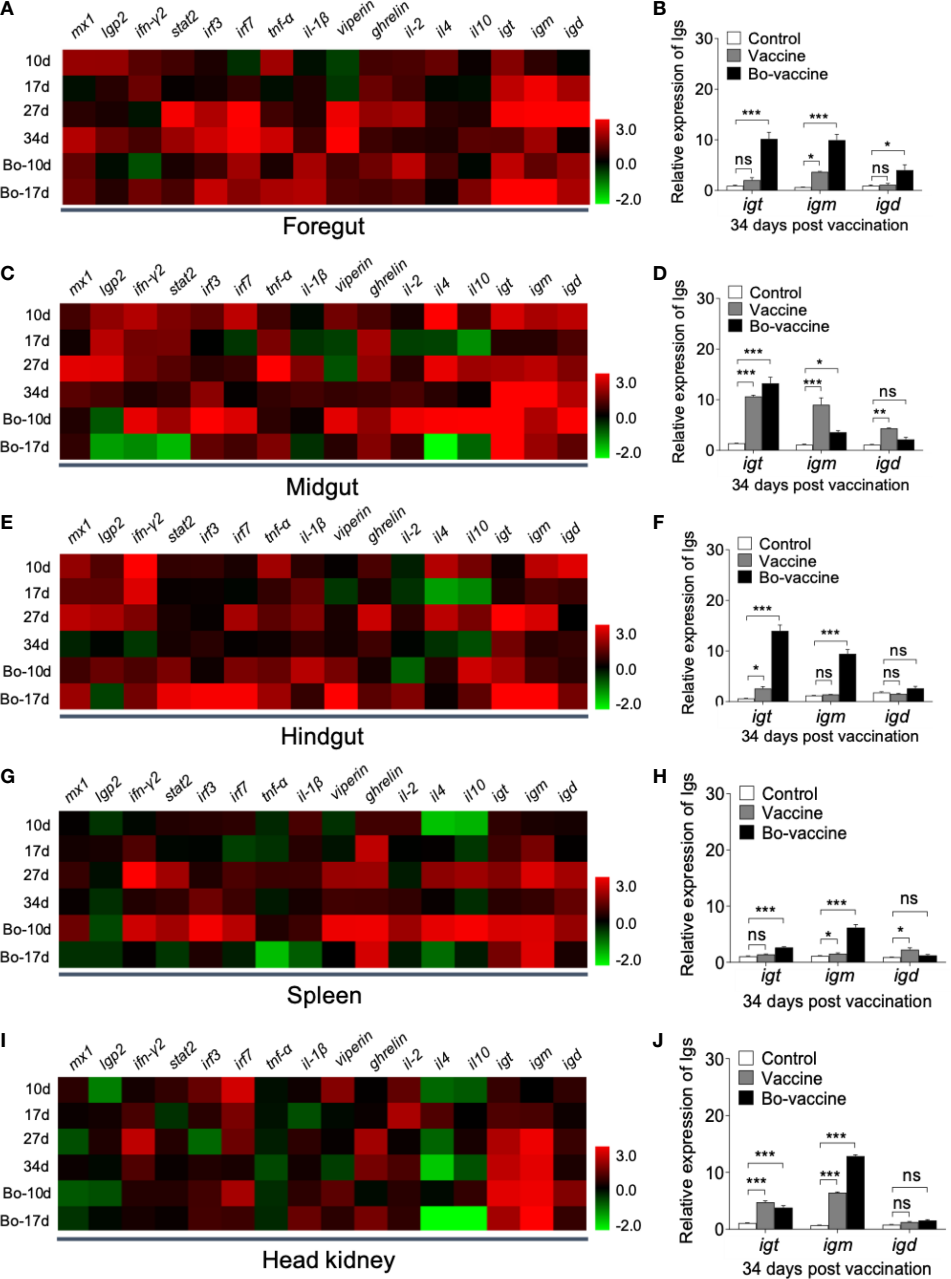
Immune responses in gibel carp vaccinated with oral administration of CyHV-2 yeast vaccine. Heat map illustrates expression profiles of immune genes in gibel carp foregut **(A)**, midgut **(C)**, hindgut **(E)**, spleen **(G)**, and head kidney **(I)** of vaccine and bo-vaccine *vs*. control fish measured at 10,17, 27, and 34 dpv (*n* = 6 fish per group). Color value: log_2_ (fold change). **(B, D, F, H, J)** Fold changes of *igt*, *igm* and *igd* in foregut, midgut, hindgut, spleen, and head kidney was detected at 34 dpv (*n* = 6 fish per group). Control *vs*. Vaccine and Bo-vaccine: ns, not significant, *P < 0.05, **P < 0.01, ***P < 0.001, one-way ANOVA with Bonferroni correction. Data are representative of three different independent experiments (mean ± SEM).

To evaluate the kinetics of the immune response in gibel carp after oral yeast vaccination, the expression of 16 immune- and antivirus-related genes including myxovirus resistance 1 (*mx1*), laboratory of genetics and physiology 2 (*lgp2*), interferon (*ifn-γ2*), signal transducer and activator of transcription (*stat2*), interferon regulatory factors (*irf3* and *irf7*), cytokines (*tnf-α* and *il-1β*), virus inhibitory protein gene (*viperin*), growth hormone-releasing peptide gene (*ghrelin*), adaptive immune -related genes (*il-2*, *il-4* and *il-10*), and immunoglobulin heavy chain genes (*igt*, *igm*, and *igd*) in mucosal (foregut, midgut, and hindgut) and systemic (head kidney and spleen) tissues were detected by qPCR at 10, 17, 27, and 34 dpv (**Figure 5**). Compared to the control group, our results showed that most of the innate immune-and antivirus-related genes increased significantly, and the expression level of these genes in the gut was higher than that in the spleen and head kidney after vaccination. Moreover, this higher expression started earlier in the gut than spleen and head kidney. The adaptive immune-related genes were significantly upregulated in the midgut, hindgut, and spleen after vaccination (**Figures 5C**, **E**, **G**). Immunoglobulins serving as receptors on B cells play a crucial role in adaptive immunity. Here, we found that expression levels of *igt*, *igm*, and *igd* were significantly upregulated in the gut (**Figures 5A**, **C**, **E**), spleen, and head kidney after vaccination (**Figures 5G**, **I**). Interestingly, the expression of the three immunoglobulin genes in the gut was different from that in systemic tissues. In the gut, the expression of *igt* in the vaccine group was much higher than that in systemic tissues compared with that of control fish (**Figures 5B**, **D**, **F**). Conversely, the expression of *igm* and *igt* both increased notably in the systemic tissues, with the increases in *igm* expression level being dominant (**Figures 5G**, **I**). Importantly, the expression levels of *igt* and *igm* both in the gut and systemic tissues were higher in the booster vaccine group than that in the vaccine group. Together, these data supported that the yeast vaccine could effectively induce innate and adaptive immune responses in both mucosal and systemic immunity of gibel carp.

### The yeast vaccine protects gibel carp from CyHV-2 infection

**Figure 6:**
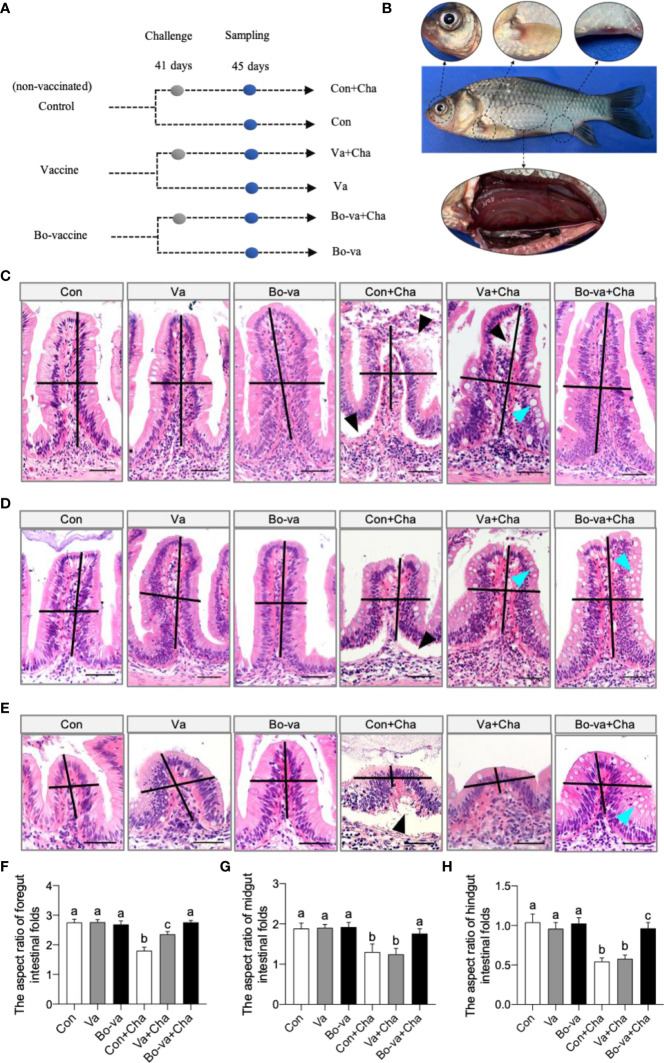
Pathological changes of gibel carp after challenge with CyHV-2 among the control group, vaccine group, and booster vaccine group. **(A)** Strategy to challenge with CyHV-2. Briefly, at 41 days, control, vaccinated, and booster-vaccinated fish were challenged by injection with CyHV-2, and at 4 dpc, the six groups of fish treatments (including: Con [control fish + PBS], Va [vaccinated fish +PBS], Bo-va [booster-vaccinated fish + PBS], Con+Cha [control fish + CyHV-2], Va+Cha [vaccinated fish + CyHV-2], Bo-va+Cha [booster-vaccinated fish + CyHV-2]) were analyzed for pathological changes of foregut, midgut, and hindgut, respectively. **(B)** The clinical observation following challenge with CyHV-2. **(C–E)** Histological examination by H&E staining of foregut, midgut, and hindgut from Con, Va, Bo-va, Con+Cha, Va+Cha, and Bo-va+Cha groups fish (*n* = 6 fish per group). Black triangle indicates epithelial cell sloughing and necrosis. Blue triangle indicates the goblet cells. Scale bars, 50 μm. **(F–H)** The length-width ratio of foregut **(C)**, midgut **(D)**, and hindgut **(E)** intestinal folds in these groups fish from C-E (*n* = 6 fish per group). Different superscript letters in each group (a, b, c) denote significant variations suggested by the Kruskal–Wallis statistics at 95% of significance, followed by the Dunn test with Bonferroni adjustment as the *post hoc* test (*p* < 0.05). Data are representative of three different independent experiments (mean ± SEM).

To further test the efficacy of the yeast vaccine against CyHV-2, the immunized fish were challenged with CyHV-2 by intraperitoneal injection at 41 dpv. Tissue samples were collected for histological analysis 4 days (45 dpv) after challenge (**Figure 6A**). Typical clinical symptoms of HVHN were observed in the Con+Cha group at 2 dpc, such as eyeball protrusion, abdominal swelling and congestion, and body surface hemorrhages (**Figure 6B**). The histological analysis showed that CyHV-2 infection caused morphological changes in the gut (**Figures 6C**–**E**). The aspect ratio of intestinal folds in the Con+Cha group and the Va+Cha group was significantly decreased 4 days after infection with CyHV-2 compared to the controls, whereas there was no difference between the control group and booster vaccine group (**Figures 6F**–**H**). Histological studies exhibited a significant increase in the number of goblet cells in the Va+Cha and Bo-va+Cha groups compared to Con+Cha fish after challenge (**Supplementary Figure 1**). Moreover, different degrees of tissue damage were observed in both Con+Cha and Va+Cha groups, including epithelial cell sloughing and necrosis (**Figures 6C**–**E**). However, the tissue injury in the gut of the Bo-va+Cha group was significantly reduced compared to Con+Cha fish. Therefore, our findings suggested that vaccination with yeast vaccine relieves the gut damage caused by CyHV-2 infection.

**Figure 7:**
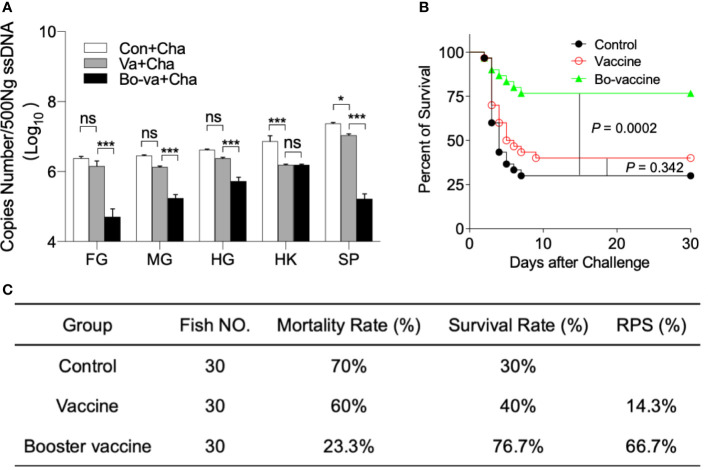
The viral load and survival rate of gibel carp after challenge with CyHV-2. **(A)** The histogram showed loads of CyHV-2 in different tissues at 4 dpc (*n* = 6 fish per group). ns, not significant, *P < 0.05, ***P < 0.001, one-way ANOVA with Bonferroni correction. Data are representative of three different independent experiments (mean ± SEM). **(B)** The cumulative mortalities of gibel carp challenged with CyHV-2. Statistical differences were evaluated by log-rank (Mantel-Cox) test. **(C)** Efficacy of yeast vaccine in gibel carp under laboratory conditions. RPS, Relative survival rate = (1 - the ratio of mortality percent in the vaccine group or booster vaccine group to in the control group) × 100%.

To further study the influence of the yeast vaccine on the viral load in the tissue, we detected the copy number of CyHV-2 in the foregut, midgut, hindgut, spleen, and head kidney in Con+Cha, Va+Cha, and Bo-va+Cha groups. As expected, CyHV-2 was detected in all selected tissues, and the viral loads in the Bo-va+Cha were significantly lower than that in the Con+Cha and Va+Cha groups, indicating the yeast vaccine could effectively inhibit the replication rate of the virus but require multiple vaccinations at least twice (**Figure 7A**). Next, we monitored the mortality of gibel carp within 30 days after the CyHV-2 challenge. All three groups of mortality appeared 2 dpc. A large number of deaths were observed in both the Con+Cha and Va+Cha groups 3 and 4 dpc, followed by fish death until 9 dpc. Importantly, upon challenge with CyHV-2, the booster vaccine group had a significantly higher survival rate (76.7%) compared with the Va+Cha (40%) and Con+Cha (30%) groups (**Figure 7B**). Although the RPS of the CyHV-2 in the vaccine group was only 14.3%, the booster vaccine effectively increased the RPS up to 66.7%. (**Figure 7C**).

## Discussion

CyHV-2 is the main viral pathogen that affects gibel carp, causing significant economic losses and severely hindering the healthy and sustainable development of the industry (29). Vaccination is undoubtedly the most appropriate approach to control various fish diseases from an efficiency, safety, environmental and ethical standpoint. Compared with the injection route, oral administration is considered to be the simplest and most cost-effective vaccination method, especially for small fish. Thus, a push toward oral delivery will be crucial to ensure the widespread adoption of CyHV-2 vaccines in the gibel carp aquaculture industry. Here, we prepared an oral yeast vaccine by surface-displaying CyHV-2 ORF25 and tested the vaccine for safety (i.e., mortality and intestinal morphological changes), immunogenicity (i.e., body fluid biochemistry indices and immune-related gene expression), and protection efficacy (i.e., intestinal pathological changes, viral load, relative survival). Our findings indicated that the vaccine is safe and effective at protecting gibel carp against CyHV-2 infection. These results demonstrated that the oral vaccine based on yeast surface-displaying is a promising candidate for preventing CyHV-2 infection.

The basic principle of a vaccine is that they stimulate the immune system to develop protective immunity without causing pathogenic effects on the host. Among the different administration methods, after oral vaccination, besides mortality, intestinal morphological changes and further damage on the intestine should be monitored to demonstrate safety. Yeast has been reported to improve animal growth when used as a carrier for oral vaccines (30). *S. cerevisiae* is the most common host for cell surface display because it is non-toxic and has been proven to be safe for humans in clinical trials (31, 32). In our study, no fish died, and no significant tissue damage was observed in the gibel carp intestine after vaccination. Collectively, these findings indicated that the recombinant yeast vaccine prepared herein was safe and therefore *S. cerevisiae* could serve as a novel carrier for CyHV-2 oral vaccines in gibel carp. Although from the perspective of genetic engineering techniques, recombinant yeast vaccines may be considered a genetically modified organism (GMO), so far, no of the recombinant yeast vaccines approved on the market have caused side effects, as illustrated by a successful example of a recombinant yeast hepatitis B vaccine (33).

The efficacy of a vaccine is largely determined by its ability to induce an immune response. Lower vertebrates such as fish are equipped with an immune system homologous to that of mammals, which encompasses both innate and adaptive immunity (34). Innate immunity serves as the first line of defense against pathogen invasion. Complement, SOD, and lysozyme (LZM) in serum and mucus are important innate immune parameters and have often been used as indicators of disease resistance (35). The complement system constitutes an important component of the innate response in bony fish due to its role in promoting the body’s inflammatory response, eliminating apoptotic cells, and regulating adaptive immunity (36). A previous study demonstrated that the neutralizing ability of rainbow trout antibodies against infectious hematopoietic necrosis virus (IHNV) can be enhanced by the action of the complement system (37). C3 is the central component of this system and plays a key role in the activation of the complement system. In the present study, the content of C3 increased significantly in the gut mucus and serum after vaccination. Interestingly, the content of C3 in foregut mucus increased earlier than that in midgut and hindgut mucus. A similar result has also been reported in the serum of gibel carp after immunization with DNA vaccine ORF25 with molecular adjuvant CCL35.2 (38). SOD is a key antioxidant enzyme and several studies have demonstrated that antioxidant capacity is positively correlated with the immune levels in fish (39, 40). Compared with the control group, the TSOD activity of immunized fish was significantly higher in our study. This result is consistent with another study that reported increases in TSOD activity in response to an inactivated viral vaccine in gibel carp (38). LZM is an important defense component of the innate immune system, which plays a crucial role in mediating protection against bacterial and viral invasion (41, 42). A previous study reported that LZM activity in the serum and intestinal mucus of grass carp increased significantly after immunization with an oral DNA vaccine compared to control fish (43). In our study, the LZM activity of the immunized fish was significantly higher in the serum, foregut, and hindgut mucus. Interestingly, however, there was no significant change in the midgut after vaccination. A recent study on the effect of an oral microencapsulated vaccine in grass carp showed that compared to the unvaccinated fish, the fish vaccinated with the oral vaccine exhibited significant up-regulation in the activity of C3, TSOD, and LZM (44), which was very similar to our results. These results indicated that EBY100-pYD1-ORF25 can enhance the nonspecific immune system in gibel carp. The increase in the activity of these indicators may be due to an increase in the number of cells involved in this process, such as migration of head kidney leukocytes, or enhanced pathogen resistance (45). However, the exact mechanisms involved need to be further explored.

Additionally, the expression of several immune- and antiviral-related genes was measured in both mucosal tissue (i.e., gut) and systemic organs (i.e., spleen and head kidney). As expected, a significant upregulation of these genes was detected after vaccination. Interferon (IFN) is the first line of defense against viral infection in vertebrates (46–48). IFN exerts its antiviral effects by inducing the expression of interferon‐stimulated genes (ISGs), including *mx1*, *viperin*, *irf3*, and *irf7*, among others (49, 50). The transcript levels of these genes were significantly increased after infection with the virus or treatment with poly I:C (51–54). Cytokines, which act as modulators of the immune responses, are related to both innate and adaptive immune responses. Particularly, pro-inflammatory cytokines such as interleukin-1β and TNF-α can limit viral propagation and viral protein expression by triggering inflammatory cytokine storms, enhancing macrophage respiratory burst activity, and inducing apoptosis (55). LGP2, an important member of the RIG-I-like receptor (RLRs) family, is known to play an essential role in antiviral responses (56). In this study, the expression of these genes was considerably induced in the gut and systemic organs after vaccination. Compared with intestinal mucosal immunity, system immunity lags, indicating that part of the antigen might be transported to systemic organs or that immune effector cells enter the blood circulation *via* venules of the lamina propria. Importantly, these genes were more upregulated in the gut than in the spleen and head kidney, indicating that the immunostimulatory effects of the oral vaccine were stronger in the gut than in the systemic organs. It was proposed that the structure and function of teleost IFN-γ2 are similar to mammalian IFNγ, which is a typical Th1 immune response cytokine, that elicited high antiviral activity (46–48). Here, the expression of *ifn-γ2* was considerably induced in the gut at an early stage, especially in the hindgut. In fish, enterocytes and intraepithelial macrophages have been shown to play a role in antigen uptake in the second gut segment (57). A previous study has shown that with oral delivery of recombinant *S. cerevisiae* cells to *C. auratus*, EGFP expression in the hindgut was detected at 5 days post the first vaccination. Furthermore, specific serum antibodies were detected in vaccinated fish, which may demonstrate that yeast cells could be engulfed by the intestinal epithelial cells of fish and that the incorporated plasmid passed across the gut barrier, allowing the transcript to be efficiently expressed (58). Rombout et al. showed that there are differences in the distribution of antigen-presenting cells (APCs) between different intestinal segments, with more APCs in the second segment than in the first (59, 60). Therefore, yeast surface display technology may be an ideal vaccine delivery system that can deposit antigen in the second intestinal segment. Overall, these results suggested that the oral yeast vaccine prepared herein can enhance immune responses, especially in the intestine, to establish a barrier against viral infection. Relevant conclusions also exist in oral vaccines against bacterial and parasitic infections (61–63). More importantly, our findings indicated that these genes could be rapidly upregulated after booster vaccination. It is worth noting that growth hormone-releasing peptide (ghrelin), a brain intestinal peptide, is an important appetite-increasing factor (64). Therefore, we examined *ghrelin* expression in gibel carp to determine whether the yeast vaccine would affect its feeding rates. High *ghrelin* expression levels were detected after vaccination, suggesting that the oral yeast vaccine was highly palatable for gibel carp.

Similar to mammals, teleost fish also possess adaptive immunity and immunological memory (65). Vaccines utilize adaptive immunity and memory by exposing the body to antigens. Therefore, an enhanced immune response can be rapidly activated when live pathogens invade the body, thus protecting the host from pathogen infection. IL-2 is a family of polypeptides that promote the differentiation and proliferation of B cells induced by CD4^+^ Th cells, in addition to enhancing the activity of NK cells, thereby improving the level of cellular antiviral immunity (66). IL-4 and IL-10 can promote the differentiation and proliferation of T cells, which in turn regulates B cell differentiation and antibody secretion (67, 68). Immunoglobulins (Igs) are important components of adaptive immunity. Teleost B cells are known to produce three different Ig types (IgM, IgD, and IgT), but only IgT and IgM can elicit specific immune responses to pathogens (69). Our study found that the expression of *igt* and *igm* was significantly upregulated both in the gut and systemic tissues (i.e., spleen and head kidney) after vaccination. Similar to the previous study (70), here the expression of *igt* was much higher than that of *igm* in the gut, and in systemic tissues, the expression level of *igm* was significantly higher than that of *igt*. Moreover, similar to our study, the expression of *igt* in the hindgut was significantly stronger than that in the spleen or head kidney after vaccination with an oral vaccine against IHNV (71), virus-specific IgM and IgT were found to be significantly elevated in systemic and mucosal tissues, respectively, in rainbow trout after infection with IHNV (72). In addition to the potential complementary role of IgD to canonical mucosal IgT and IgM responses, IgD may establish a mutualistic relationship with commensals to enhance mucosal homeostasis (73). Some studies have demonstrated that IgD acts as an Ag-binding receptor and plays an important role in innate immunity (74, 75). In our study, *igd* expression was significantly elevated in the midgut and hindgut 10 days after vaccination, suggesting that IgD may have some antivirus function at the early stages of infection. Notably, the expression of adaptive immune-related genes after booster vaccination was significantly higher than after primary vaccination. Overall, our results showed that the oral yeast vaccine could effectively elicit innate and adaptive immune responses both in mucosal and systemic tissues, thereby protecting gibel carp against CyHV-2 infection.

To evaluate the protective effects of the vaccine, gibel carp were challenged with CyHV-2 *via* injection. After the challenge, a large number of control fish exhibited the typical clinical signs of HVNV, including dorsal fin bleeding, abdominal congestion, and eyeball protrusion (4). In a histological assay, we found that the most severe lesions including sloughing of epithelial cells and lower aspect ratio of intestinal folds were observed in the control fish after 2 dpc. A moderate pathological change was found in the fish that were vaccinated only once. In contrast, no significant changes were observed in the gut mucus epithelial cells or the intestinal folds aspect ratio of the booster-vaccinated fish. Mucus protects and lubricates the epithelium and is part of the innate defense system that protects fish against pathogens (76). The fish intestinal epithelium is interspersed with mucus-producing goblet cells, which play a crucial role in protecting the intestinal barrier and can produce antimicrobial proteins, chemokines, and cytokines in innate immunity (77). A previous study has demonstrated that a significant increment in the goblet cell density was observed for autolyzed yeast in the *Sparus aurata* diet (78). Here, gibel carps significantly increased the number of intestinal goblet cells in both the vaccine and booster vaccine groups after being challenged with the CyHV-2, which may correlate with their survival and recovery. Moreover, the CyHV-2 viral load in the gut, spleen, and head kidney was significantly lower in the vaccinated fish, especially in the booster-vaccinated fish, than in the non-vaccinated fish. The absence of pathological changes coincided with the improvement in the survival of vaccinated fish, especially those vaccinated twice, achieving a relative percent survival (RPS) rate of 14.3% in the vaccine group and 66.7% in the booster vaccine group. In turn, this suggested that the protective effect of booster vaccination is stronger than that of single vaccination. Previous studies have demonstrated that primary oral vaccination does not induce a strong immune response, whereas booster oral vaccination induces a strong secondary immune response and significantly improves the survival of fish (63, 79). It is worth noting that the presence of a strong adjuvant is required for oral vaccine preparations to improve disease resistance (63). In a previous study, gibel carp injected with a recombinant baculovirus vectored vaccine achieved an RPS of 80.01% upon challenge with CyHV-2 (11). Although the yeast vaccine is slightly less protective than some injectable vaccines, it is still highly promising due to the unique advantages of the oral administration route. Although it is well known that yeast has immunostimulatory effects (17), previous studies have shown that feeding yeast alone did not induce significant differences in the antivirus gene expression and RPS compared to controls (24, 30, 70). Nevertheless, we cannot be ruled out the possibility that its presence alone (non-recombinant-yeast) in the intestinal tract affects the protection of this vaccine in the present study. Moreover, it’s reported that commensal yeast likely plays a significant part in the early development of fish larvae (80). Therefore, future studies need to clarify the effect of feeding yeast alone on antiviral infection of gibel carp.

In conclusion, the orally-administered yeast vaccine developed in this study was deemed relatively safe and elicited strong innate and adaptive immune responses in both mucosal and systemic tissues. More importantly, the vaccine remarkably improved the survival rate of gibel carp against CyHV-2 infection by relieving intestinal injury and inhibiting viral replication, which results in a significant increase in RPS rates. Although the protective effect of booster vaccination is better than that of single vaccination, further research is required to assess the long-term efficacy of these treatments, while the addition of potentiators as adjuvants requires further investigations. Whatever the case, the present results suggested that the oral yeast vaccine developed in this study is a promising candidate for controlling CyHV-2 in gibel carp farming.

## Data availability statement

The original contributions presented in the study are included in the article/**Supplementary Material**. Further inquiries can be directed to the corresponding author.

## Ethics statement

The animal study was reviewed and approved by The Animal Experiment Committee of Institute of Hydrobiology, Chinese Academy of Sciences.

## Author contributions

Z-RD and Q-JM performed most of the experiments and wrote the manuscript. Z-RD and W-GK analyzed the data. YZ, D-CQ, X-YW, and G-FC helped with most of the experiments. ZX designed the experiments and revised the manuscript. All authors contributed to the article and approved the submitted version.

## Funding

This work was supported by grants from the National Natural Science Foundation of China (U1905204, 32073001, and 31873045) and a grant from the Key Laboratory of Sichuan Province for Fishes Conservation and Utilization in the Upper Reaches of the Yangtze River, Neijiang Normal University (NJTCSC01).

## Acknowledgments

We thank Dr. Yong-Zhou (Yangtze River Fisheries Research Institute of Chinese Academy of Fishery Sciences) for his generous gifts of CyHV-2.

## Conflict of interest

Author Y-ZL and T-SA was employed by the company Wuhan Chopper Fishery Bio-Tech Co., Ltd.

The remaining authors declare that the research was conducted in the absence of any commercial or financial relationships that could be construed as a potential conflict of interest.

The reviewer NW declared a shared affiliation with the authors ZX and WK to the handling editor at the time of review.

## Publisher’s note

All claims expressed in this article are solely those of the authors and do not necessarily represent those of their affiliated organizations, or those of the publisher, the editors and the reviewers. Any product that may be evaluated in this article, or claim that may be made by its manufacturer, is not guaranteed or endorsed by the publisher.
