# Supplementary material for: Gut mucosal immune responses and protective efficacy of oral yeast Cyprinid herpesvirus 2 (CyHV-2) vaccine in Carassius auratus gibelio
Source: Front Immunol. 2022 Jul 29;13:932722. doi: 10.3389/fimmu.2022.932722 (PMC9373009; doi:10.3389/fimmu.2022.932722)
Supplement: Supplementary file 1 [file DataSheet_1.docx]

**Gut mucosal immune responses and protective efficacy of oral yeast Cyprinid herpesvirus 2 (CyHV-2) vaccine in *Carassius auratus gibelio***

Zhao-Ran Dong^1¶^, Qing-Jiang Mu^1¶^, Wei-Guang Kong^2^, Da-Cheng Qin^1^, Yong Zhou^3^, Xin-You Wang^1^, Gao-Feng Cheng^1^, Yang-Zhi Luo^4^, Tao-Shan Ai^4^, and Zhen Xu^2*^

^1^Department of Aquatic Animal Medicine, College of Fisheries, Huazhong Agricultural University, Wuhan, Hubei 430070, China

^2^State Key Laboratory of Freshwater Ecology and Biotechnology, Institute of Hydrobiology, Chinese Academy of Sciences, Wuhan, Hubei, 430072, China; Laboratory for Marine Biology and Biotechnology, Qingdao National Laboratory for Marine Science and Technology, Qingdao 266071, China

^3^Yangtze River Fisheries Research Institute, Chinese Academy of Fishery Sciences, Wuhan, Hubei 430223, China

^4^Wuhan Chopper Fishery Bio-Tech Co., Ltd, Wuhan Academy of Agricultural Science, Wuhan, 430207, China

^¶^ These authors contributed equally to this work.

^*^Corresponding Author: zhenxu@ihb.ac.cn.

**Supplementary materials**

**FIGURE** **S1**

**FIGURE** **S1** | The numbers of goblet cells per intestinal fold in foregut, midgut, and hindgut tissue of Con+Cha, Va+Cha, and Bo-va+Cha groups fish (*n* = 6 fish per group). Con+Cha vs. Va+Cha and Bo-va+Cha: ns, not significant, **P* < 0.05, ****P* < 0.001, one-way ANOVA with Bonferroni correction. Data are representative of three different independent experiments (mean ± SEM).

**FIGURE** **S2**


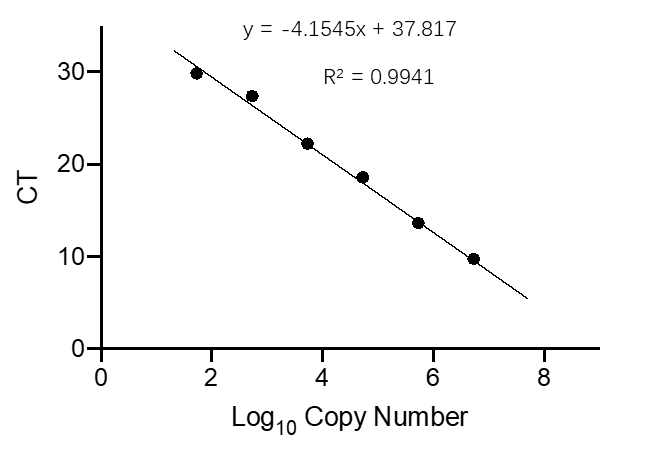


**FIGURE** **S2** | Standard curve for CyHV-2 copy number vs cycle threshold (Ct) value.

**TABLE S1 |** Gene-specific primers used for qPCR in this study.

| **Gene** | **Primer Sequence (5’- 3’)** | | **GenBank**  **accession no.** |
| --- | --- | --- | --- |
|  | **Forward primer** | **Reverse primer** |  |
| *mx1* | CCTGACCTCACTCTCATCG | CCTAAAGTCCTTTCGCCCTC | AY303813.1 |
| *lgp2* | TAAAGGCAATGATGTCGATGATT | GCAATGTGCGTTCCAAACG | XM026262921.1 |
| *ifn-γ2* | TCCCTGAGAACCTGGACAAGA | TTCTGCATGCGAGTGAATATCC | JX657682.1 |
| *stat2* | GCTGCTCAATGCACTCGATA | CCACTTCCAGTCCACCAGTT | XM026259725.1 |
| *irf3* | TCCAGGCCAAGCATACGAA | CCATTTGCAACAGCCATCAT | HQ229991.1 |
| *irf7* | CAACGAGCACCCTAACGA | CCACCTGGCTGAGCAATT | AY177629.1 |
| *tnf-α* | CGCTACTCTGATTCCTATGGC | GCTTTCGCTGTTGCCTTTCT | KF500408.1 |
| *il-1β* | TTTGTGAAGATGCGCTGCTC | CCAATCTCGACCTTCCTGGTG | AB757758.1 |
| *viperin* | AAGTCATAGGTCGAGGTCAGGG | CGTTTTCACCTTCAATTAGCAGA | AY303809.1 |
| *ghrelin* | TTCATGATGAGTGCTCCGTTC | GTCAGAATTCAAGTGGCGAATC | HM567312.1 |
| *il-2* | GACCACAAAGGTAGACCCATCC | GAGGTTTGTGCGGAATGGAC | MN338056.1 |
| *il-4* | CGATTGTAGCCGTTACTGGGT | TGGCAAATGTGTTCCTCCG | KX574595.1 |
| *il-10* | AGCCATGGGAGAGCTTGATA | ATGATGACGTGCAAGCGTT | HQ259106.1 |
| *igt* | ATTCATTGTCAGACCTTCACTCAG | GCAGTGTCTTCAGTCTTGAGGCT | GQ201446.1 |
| *igm* | AGCTCAACCATCTGCACCAA | ATGTAAGCGAGTCCGCAGGT | GU563726.1 |
| *igd* | TGGAACAAGAACACAGGC | ATTATTGGAGGAACGCAG | Not available |
| *β-actin* | TGAAGATCCTGACCGAGCGT | GGAAGAAGAGGCAGCGGTTC | LC382464.1 |
| *cyhv-2* | AGCCCAAGATCAATCACAAT | CCAGACAGCCTTCAAACAC | NC019495.1 |
